# Determination of Tangeretin in Rat Plasma: Assessment of Its Clearance and Absolute Oral Bioavailability

**DOI:** 10.3390/pharmaceutics10010003

**Published:** 2017-12-29

**Authors:** Mai Gamal Elhennawy, Hai-Shu Lin

**Affiliations:** Department of Pharmacy, Faculty of Science, National University of Singapore, 18 Science Drive 4, Singapore 117543, Singapore; mai.gamal@u.nus.edu

**Keywords:** tangeretin, high performance liquid chromatography, clearance, absolute oral bioavailability

## Abstract

Tangeretin (TAN) is a dietary polymethoxylated flavone that possesses a broad scope of pharmacological activities. A simple high-performance liquid chromatography (HPLC) method was developed and validated in this study to quantify TAN in plasma of Sprague-Dawley rats. The lower limit of quantification (LLOQ) was 15 ng/mL; the intra- and inter-day assay variations expressed in the form of relative standard deviation (RSD) were all less than 10%; and the assay accuracy was within 100 ± 15%. Subsequently, pharmacokinetic profiles of TAN were explored and established. Upon single intravenous administration (10 mg/kg), TAN had rapid clearance (*Cl* = 94.1 ± 20.2 mL/min/kg) and moderate terminal elimination half-life (*t*_1/2 *λz*_ = 166 ± 42 min). When TAN was given as a suspension (50 mg/kg), poor but erratic absolute oral bioavailability (mean value < 3.05%) was observed; however, when TAN was given in a solution prepared with randomly methylated-β-cyclodextrin (50 mg/kg), its plasma exposure was at least doubled (mean bioavailability: 6.02%). It was obvious that aqueous solubility hindered the oral absorption of TAN and acted as a barrier to its oral bioavailability. This study will facilitate further investigations on the medicinal potentials of TAN.

## 1. Introduction

**Figure 1:**
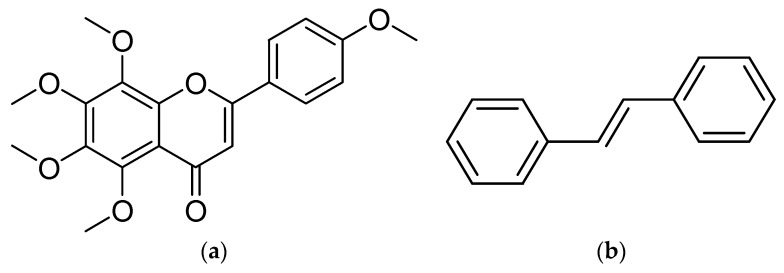
Chemical structures of: (**a**) Tangeretin; (**b**) *trans*-Stilbene (internal standard).

Tangeretin (4′,5,6,7,8-pentamethoxyflavone, TAN, Figure 1a) is one of the most abundant polymethoxyflavones present in the peel of various citrus fruits [1,2]. During the past two decades, its health-promoting effects including anti-diabetic [3,4,5], anti-inflammatory [6,7,8,9,10,11,12], anti-oxidant [6,9], anti-viral [13], cardio-protective [14], hepato-protective [7,15], nephro-protective [9] and lipid lowering [16] have been reported in various pre-clinical studies. Besides its intrinsic anti-cancer activities (both chemoprevention and chemotherapy) [17,18], as a potent inhibitor of various efflux transporters [19,20], co-administration of TAN was found to sensitize various cancer cells to chemotherapy/radiotherapy [20,21,22]. Moreover, its therapeutic potentials in some neurodegenerative disorders including Alzheimer’s disease [23,24], epilepsy [25] and Parkinson’s disease [24,26,27] have been observed. Clearly, TAN is a promising dietary flavone that warrants further medicinal exploration.

Pharmacokinetic profiling is an integral step in drug discovery and development. The physiological reference of the in vitro pharmacological effects of a drug candidate depends on its in vivo pharmacokinetic profile. Therefore, the measurement of the level of a drug candidate and its active metabolite in biological matrices over time helps to build a better correlation between its dosing regimen and its pharmacological responses. Although the pharmacokinetic profiling of TAN has been attempted before [27,28,29], due to the lack of an intravenous pharmacokinetic study, many important kinetic parameters including clearance (*Cl*), apparent volume of distribution, terminal elimination half-life (*t*_1/2 *λz*_), mean residence time (*MRT*) and absolute oral bioavailability (*F*) remained unknown. Moreover, unfavorable oral pharmacokinetic profile of TAN was observed in the previous studies [27,30] although the underlying mechanisms have not been fully disclosed. Therefore, it is of interest to identify whether aqueous solubility is a barrier to the oral absorption of TAN.

In this study, an accurate and precise high-performance liquid chromatography (HPLC) method was established to measure TAN in plasma of Sprague-Dawley rats. The pharmacokinetic profiles of TAN were subsequently examined after respective single intravenous and oral administration. To our knowledge, this is the first assessment on the intravenous pharmacokinetics and *F* of TAN. The results generated from this study will facilitate further investigations on the medicinal potentials of TAN.

## 2. Materials and Methods

### 2.1. Materials and Reagents

Tangeretin (4′,5,6,7,8-pentamethoxyflavone; purity > 98%, TAN, 1, Figure 1a) was purchased from Ontario Chemicals Inc. (Guelph, ON, Canada). *Trans*-stilbene (purity > 96%, internal standard, Figure 1b) was purchased from Sigma-Aldrich. 2-Hydroxypropyl-β-cyclodextrin (HP-β-CD, degree of substitution about 0.6) and randomly methylated-β-cyclodextrin (RM-β-CD) were generously donated by Wacker (Burghausen, Germany). Sodium salt of carboxymethylcellulose (CMC) was purchased from Sigma-Aldrich (St. Louis, MO, USA). HPLC/Spectro-grade acetonitrile was purchased from Tedia (Fairfield, OH, USA). Analytical grade dimethyl sulfoxide (DMSO) was purchased from MP Biomedicals (Solon, OH, USA). Purified water (18.2 MΩ·cm at 25 °C) was generated by a Millipore Direct-Q ultrapure water system (Billerica, MA, USA) and used to prepare dosing solution as well as mobile phase throughout the study.

### 2.2. High Performance Liquid Chromatography

For the quantitation of TAN in Sprague-Dawley rat plasma, a Shimadzu (Kyoto, Japan) 2010A Liquid Chromatography was used. This HPLC apparatus is made up of an online degasser, a quaternary gradient low-pressure mixing pump, a column oven, an auto-sampler, a dual-wavelength UV-Vis detector and a system controller. The system is controlled by a personal computer using Shimadzu Class-VP Version 6.12 SP1 software (Shimadzu, Kyoto, Japan). For analysis of chromatographic data, the same software was used. Chromatographic separation was achieved using a reversed phase HPLC column (Agilent Zorbax Eclipse Plus C18: 250 × 4.6 mm i.d., 5 µm), protected by a guard column (Agilent Zorbax Eclipse Plus C18: 12.5 × 4.6 mm i.d., 5 µm) through gradient delivery of a mixture of acetonitrile and water for 13.5 min, at a flow rate of 1.5 mL/min at 55 °C. The gradient schedule was: (a) 0–3.5 min, acetonitrile: 35%; (b) 3.5–6.5 min, acetonitrile: 35–90%; (c) 6.5–10 min, acetonitrile: 90%; (d) 10–13.5 min, acetonitrile: 35%. UV absorbance at 322 nm was used to measure TAN while 312 nm was used as a reference.

### 2.3. Preparation of Samples

Stock solutions of TAN (2 mg/mL) were prepared using DMSO, then they were stored at room temperature (25 °C). In order to prepare calibration standards and quality control (QC) samples, the stock solution was diluted with pooled Sprague-Dawley rat plasma to achieve the required pre-planned concentrations of calibration standards, which were 15, 30, 90, 120, 300, 900 and 2250 ng/mL as well as QC samples, which were 45, 450 and 1200 ng/mL. The internal standard (IS) stock solution (150 μg/mL) was prepared using acetonitrile as a solvent and then frozen at −80 °C until needed for sample processing. To use IS for sample processing, stock solution was thawed then a working solution of IS was prepared by further dilution of the stock solution to 300 ng/mL using acetonitrile. During sample processing (clean up employing protein precipitation method), three volumes of IS working solution were added to one volume of rat plasma then vigorously vortexed and centrifuged at 10,000 *g* and 4 °C for 10 min. The supernatant was carefully transferred to glass auto-sampler vial. During HPLC analysis, 75 µL supernatant was injected into the HPLC system.

### 2.4. Assay Validation

This HPLC method was validated by assuring its selectivity, sensitivity, linearity (*R*^2^), precision (intra- and inter-day variation), accuracy, and the stability profiles of TAN [31,32,33]. The selectivity was assessed by comparing the chromatograms of blank plasma from 6 different rats and the corresponding plasma samples spiked with TAN and IS. The selectivity of this HPLC method was re-confirmed by analyzing the samples from our real pharmacokinetic study, i.e., chromatographic comparison between pre-dosing and post-dosing plasma samples [31,32,33]. By definition, the lower limit of quantification (LOQ) is the minimal concentration producing a signal to noise ratio not less than 5 with acceptable accuracy and precision (mean analytical recovery: 80–120%; relative standard deviation (RSD) ≤ 20%). The assay sensitivity is represented by lower LOQ. Analytical response is the ratio between peak area of TAN and IS by definition and it is used to plot the calibration curve. Linear regression was executed by GraphPad Prism Version 6 (La Jolla, CA, USA), where *x* was the concentration of TAN, *y* was the analytical response, and 1/*x*^2^ was used as a weighting factor [34]. Linearity was assessed using calibration standards at the concentrations of 15, 30, 90, 120, 300, 900 and 2250 ng/mL. The calibration curves were carried out over 5 consecutive days. For intra-day precision, 5 replicate samples were analyzed; while for inter-day precision, duplicate samples were measured. The assay precision (RSD), accuracy (%) and absolute recovery (%) were tested at 3 different concentrations of the QC samples (low QC: 45 ng/mL, medium QC: 450 ng/mL, and high QC: 1200 ng/mL). The stability profiles of TAN were investigated. Stock solution stability was tested after storage at room temperature (25 °C) for 7 days. The stability of TAN in Sprague-Dawley rat plasma under the conditions that might be encountered during sample collection, storage, processing and HPLC analysis was investigated at low QC, medium QC and high QC concentrations. Such investigations included short-term stability (at 4 °C for 24 h), long-term stability study (at −40 °C for 14 days), stability after three freeze-thaw cycles (−80 °C to 25 °C) and post-preparative stability study (at 25 °C for 24 h). The stability of TAN was not considered as an issue if its analytical response did not deviate from freshly prepared QC samples (low, medium, high) by more than 15%.

### 2.5. Pharmacokinetic Examination

This in vivo pharmacokinetic study was carried out with strict adherence to the national guidelines of Singapore and the animal handling protocol had been reviewed and approved by the Institutional Animal Care and Use Committee of the National University of Singapore (NUS) (Protocol No.: R15-1273). All animal experiments were performed in a specific pathogen-free animal facility (temperature: 22 ± 1 °C; humidity: 60–70%). Sprague–Dawley rats (male, 314 ± 15 g) were purchased from *InVivos* (Singapore) through Comparative Medicine, NUS. The rats were sheltered under a 12 h light-dark cycle with unlimited access to food and water. On the day before the pharmacokinetic study, a catheter (polyethylene tube, i.d. = 0.580 mm, o.d. = 0.965 mm, Becton Dickinson, Sparks, MD, USA) was introduced into the right jugular vein through surgery under isoflurane anesthesia. Intravenous injection of TAN and collection of blood samples were carried out via this catheter. To prevent blood clotting and sample contamination, 0.3 mL heparin-saline (10 international unit/mL) was infused into the catheter after each intravenous drug administration and blood withdrawal. This reliable model has been commonly applied in our laboratory for pre-clinical pharmacokinetic investigations of various natural products [34,35,36,37,38].

A total of 16 rats were divided into three groups. Group 1 (*n* = 5) received a single bolus intravenous injection of TAN formulated with 0.3 M HP-β-CD solution (TAN: 5 mg/mL) at the dose of 10 mg/kg, serial blood samples were collected before dosing and at 5, 15, 30, 60, 90, 120, 180, 300, 480 and 720 min post-administration. Group 2 (*n* = 6) received a single oral dose of TAN suspended in 0.3% CMC at a concentration of (TAN: 10 mg/mL) through oral gavage at the dose of 50 mg/kg, serial blood samples were collected before dosing and at 15, 30, 45, 60, 90, 120, 180, 300, 480 and 720 min post administration. Group 3 (*n* = 5) received a single oral dose of TAN solution, solubilized in 0.3 M RM-β-CD solution (TAN: 10 mg/mL) at the dose of 50 mg/kg and the blood sampling time was the same as the group 2. At each time point, 150 µL of blood was collected. The harvested plasma was frozen at −80 °C until sample preparation.

All pharmacokinetic parameters were assessed by WinNonlin standard version 1.0 (Scientific Consulting Inc., Apex, NC, USA). Following intravenous injection, the plasma TAN level dropped through a typical bi-exponential process, i.e., a quick distribution phase followed by a prolonged terminal elimination phase. Therefore, the intravenous plasma concentration-time data was fitted into the classical two-compartment first-order open model (*C = A·e^−α·t^ + B·e^−β·t^*), using a nonlinear least squares approach with a weighting factor of 1/*y*^2^ [32,33]. Other major pharmacokinetic parameters including the plasma exposure (area under the plasma concentration-time curve (*AUC*)), terminal elimination half-life (*t*_1/2_ *_λz_*), mean residence time (*MRT*), and clearance (*Cl*) were all calculated by non-compartmental method. The mean absorption time (*MAT*) in Group 2 and 3 was calculated as: *MAT* = *MRT_oral_* – *MRT_iv_*. The absolute oral bioavailability (*F*) was calculated as: *F* = (*AUC_oral_* ÷ *Dose_oral_*) ÷ (*Mean AUC_iv_* ÷ *Dose_iv_*) × 100%.

### 2.6. Statistics

All statistical calculations were conducted with GraphPad InStat version 3.10 (GraphPad Software, Inc., La Jolla, CA, USA, 2003). Since the time to maximal concentration (*t_max_*) observed in a real pharmacokinetic study is dependent on the sampling schedule and it is non-continuous data, Mann-Whitney test was chosen to compare the *t_max_* data between Groups 2 and 3. A two-tailed Chi-square test was applied to analyze a contingency table. For the other pharmacokinetic parameters obtained from Groups 2 and 3, their distribution was assessed with the Kolmogorov-Smirnov test. Since their distribution all fulfilled Gaussian distribution, a two-tailed unpaired *t*-test was selected to compare their difference. When a *p* value less than 0.05 is observed, the difference between two groups is considered to be statistically significant. Such statistical protocol has been extensively used in our recent pharmacokinetic studies [31,36,37].

## 3. Results and Discussion

### 3.1. Assay Validation

**Figure 2:**
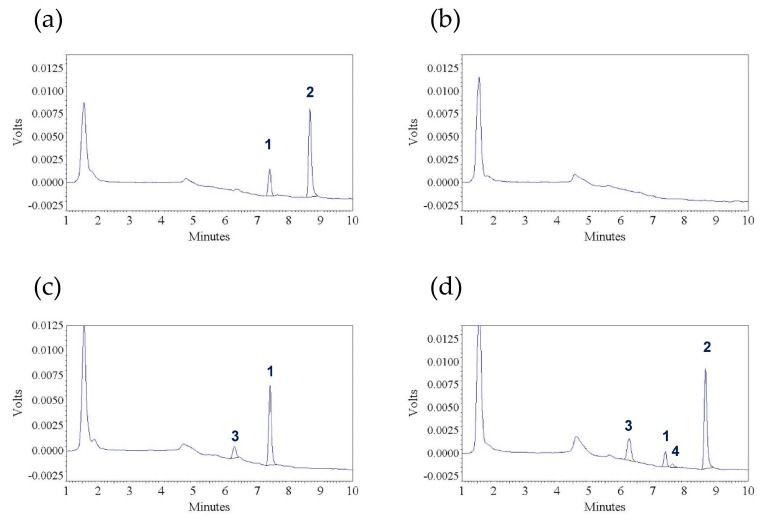
Representative chromatograms (UV absorbance, λ = 312 nm) of: (**a**) a blank plasma sample spiked with tangeretin (300 ng/mL) and *trans*-stilbene (internal standard) (900 ng/mL); (**b**) a pre-dosing plasma sample; (**c**) a post-dosing plasma sample collected from a Sprague—Dawley rat at 30 min after receiving an intravenous dose of tangeretin (10 mg/kg) (without internal standard); and (**d**) a post-dosing plasma sample collected from a Sprague—Dawley rat at 30 min after receiving an oral solution of tangeretin (50 mg/kg) (with internal standard). Peak 1: tangeretin, peak 2: *trans*-stilbene (internal standard), peaks 3 and 4: unidentified metabolites.

This HPLC method was validated by assuring its selectivity, sensitivity, precision, accuracy, and the stability profiles of TAN. The selectivity of this assay was confirmed. Under our chromatographic conditions, TAN (peak 1) and IS (peak 2) were completely separated and eluted at ~7.4 and 8.7 min respectively (Figure 2a). No notable interference peak was identified in the chromatograms of either blank plasma samples (*n* = 6) or pre-dosing plasma samples (*n* = 16). A representative chromatogram of a pre-dosing sample is shown in Figure 2b. Moreover, TAN and IS were well separated from the metabolites (peaks 3 and 4) as shown in the chromatograms acquired from post-dosing samples (Figure 2c,d).

The lower LOQ of TAN was found to be 15 ng/mL. All calibration curves were linear (*R*^2^ > 0.999). The accuracy of this HPLC assay was tested at low, medium, and high concentrations with the QC samples and excellent accuracy was observed (Table 1). This HPLC method also displayed good precision as the intra-day or inter-day RSDs were all less than 10% (QC data are shown in Table 1). Similarly, the simple plasma clean-up procedure resulted in an appropriate absolute recovery rates (Table 1). The stability profiles of TAN were assessed at different experimental conditions and the results are shown in Table 2. The stability profiles of TAN were quite good.

Clearly, a rapid HPLC method has been established for the measurement of TAN in rat plasma. This straightforward method enabled a pre-clinical pharmacokinetic assessment of TAN in Sprague–Dawley rats.

### 3.2. Application to a Pre-Clinical Pharmacokinetic Study

**Figure 3:**
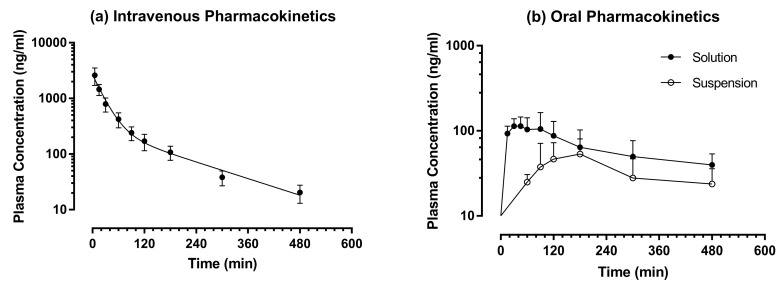
Pharmacokinetics of tangeretin. Symbols represent mean values while error bars represent SD. Intravenous: *n* = 5 except at 5 min, where *n* = 4; Oral solution (50 mg/kg): *n* = 5 except at 480 min where *n* = 3; Oral suspension (50 mg/kg): *n* = 6 except at 60 and 480 min where *n* = 3, at 90 and 120 min, where *n* = 5.

Since TAN is a water-insoluble polymethoxylated flavone, 0.3 M HP-β-CD was applied to solubilize TAN into an intravenous dosage form (5 mg/mL). The intravenous pharmacokinetic profile of TAN was tested after a single bolus injection of TAN at the dose of 10 mg/kg (Group 1) and the plasma TAN versus time profile is shown in Figure 3a. Following intravenous injection, the plasma TAN concentration dropped through a typical bi-exponential process, i.e., a quick distribution phase followed by a prolonged terminal elimination phase. Hence, the intravenous plasma concentration-time data was fitted into the classical two-compartment first-order open model. The pharmacokinetic parameters of TAN are reported in Table 3. TAN possessed a moderate apparent volume of distribution of the central compartment (*V_c_* = 4.20 ± 0.82 L/kg), a rapid clearance (*Cl* = 94.1 ± 20.2 mL/min/kg) and a moderate terminal elimination half-life (*t*_1/2 *λz*_ = 166 ± 42 min). Its *Cl* value exceeded the hepatic blood flow rate, indicating the involvement of extra-hepatic elimination of TAN. At 12 h post-administration, TAN level dropped below lower LOQ (<15 ng/mL).

To identify the influence of aqueous solubility on its oral bioavailability, the oral pharmacokinetic profiles of TAN were examined using two different formulations, i.e., a suspension prepared by 0.3% CMC and a solution formulated with 0.3 M RM-β-CD. To minimize the impact of dosing volume on oral absorption, the strength of TAN was fixed at 10 mg/mL for both formulations. The oral plasma TAN versus time profiles are shown in Figure 3b. When TAN was given in a suspension formulation (Group 2, 50 mg/kg), a delayed absorption was observed, and TAN was not quantifiable in all plasma samples collected within the first 45 mins after oral gavage (<15 ng/mL). Its total plasma exposure was very low and the maximal plasma concentration (*C_max_*) was 65.3 ± 20.1 ng/mL. Among 60 post-dosing plasma samples, TAN was measurable only in 31 samples. Moreover, the plasma TAN levels were highly erratic and the time to maximal plasma concentration (*t_max_*) ranged from 90 to 720 min. To roughly estimate the magnitude of its absolute oral bioavailability (*F*), the value of TAN in those unquantifiable samples collected from 15 to 480 min in Group 2 was adjusted to the lower LOQ (15 ng/mL) although such approach would over-estimate *AUC* to a limited extent. Altogether, the plasma TAN concentration in 26 samples from 6 rats were adjusted. Such adjustment was not applicable to Groups 1 and 3. After such manipulation, the mean *F* of Group 2 was found to be < 3.05%. When TAN was given in a solution formulation (Group 3, 50 mg/kg), immediate oral absorption was observed, and TAN could be quantified in all plasma samples collected at 15 min after oral dosing, the first post-dosing time point. The *t_max_* could be achieved within 90 min and it was much shorter than the *t_max_* of Group 2 (Mann-Whitney test: *p* < 0.01). The fully solubilized dosage form of TAN not only increased the rate but also enhanced the extent of oral bioavailability. Among 50 plasma samples collected after oral dosing in Group 3, TAN was measureable in 45 samples (Group 3 vs. Group 2, Chi-square test: *p* < 0.0001). The *C_max_* and *AUC*_0→*last*_ of Group 3 were at least two-fold as high as Group 2 (two-tailed *t*-test: *p* < 0.01). Obviously, aqueous solubility was a barrier to the oral absorption of TAN and solubility enhancing excipient such as RM-β-CD was required to facilitate the delivery of TAN. Similar results have been observed with apigenin trimethyl ether (5,7,4′-trimethoxyflavone), another polymethoxyflavone in our recent study [38].

Generally, aqueous solubility, membrane permeability and metabolic stability are the key determinants of the oral bioavailability of a xenobiotic [39,40]. Although the penetration of TAN through membrane permeability has not been well examined, its physiochemical properties such as LogP value (2.7) [41], molecular weight (372.4) [42], topological polar surface area (72.4 Å^2^) [42], number of rotatable bond (6) and sum of hydrogen bond acceptor (7) [42], do not imply problematic membrane permeability. However, the metabolic stability of TAN appeared to be another major obstruction to its extensive metabolism, which had been reported in previous study [28]. Moreover, TAN has been well recognized as a P-glycoprotein substrate [19,20]. Therefore, even when TAN was given in a solution formulated with 0.3 M RM-β-CD, its mean *F* was less than 10%. Clearly, it is of interest to assess the biological activities of TAN metabolites in future studies. Besides aqueous solubility, metabolic instability appears to be another barrier to the F of TAN.

## 4. Conclusions

An accurate and precise HPLC method was established to measure TAN in plasma of Sprague-Dawley rats. The pharmacokinetic profiles of TAN were subsequently examined after respective single intravenous and oral administration. To our knowledge, this is the first assessment on the intravenous pharmacokinetics and *F* of TAN. Aqueous solubility of TAN has been found to be a barrier to its oral absorption. The results generated from this study will facilitate further investigations on the medicinal potentials of TAN.

## Figures and Tables

**Table 1:** Accuracy and precision of the high-performance liquid chromatography (HPLC) assay ^a^.

**Intraday Assay**	**Nominal Concentrations (ng/mL)**		
	**45.0**	**450**	**1200**
Measured concentrations (ng/mL)	47.1 ± 1.0	434.8 ± 6.9	1149 ± 6
Accuracy (%)	104.6 ± 2.2	96.6 ± 1.5	95.7 ± 0.5
RSD (%)	2.1	1.6	0.6
Absolute Recovery (%)	89.2 ± 3.5	105.1 ± 2.3	99.5 ± 0.86
**Inter-day Assay**	**Nominal Concentrations (ng/mL)**		
	**45.0**	**450**	**1200**
Measured concentrations (ng/mL)	44.3 ± 1.3	440 ± 9.8	1183 ± 30
Accuracy (%)	98.5 ± 2.9	97.7 ± 2.1	98.8 ± 2.4
RSD (%)	3.0	2.3	2.5

^a^ Results are presented as Mean ± SD (*n* = 5); RSD: relative standard deviation.

**Table 2:** Stability Profiles of Tangeretin ^a^.

Storage Conditions	Remaining (%)		
	Nominal Concentration (ng/mL)		
	45.0	450	1200
Plasma samples stored for 24 h at 4 °C	101.7 ± 4.5	103.2 ± 4.6	94.9 ± 1.9
Post-preparative samples stored for 24 h at 25 °C	89.4 ± 4.0	113.1 ± 3.0	87.0 ± 2.1
Plasma samples after three freeze-thaw cycles	90.6 ± 7.1	95.2 ± 6.9	93.8 ± 0.3
Plasma samples stored for 14 days at −80 °C	93.4 ± 4.1	110.4 ± 2.6	98.5 ± 1.0
Stock solution stored for 7 days at 25 °C	111.5 ± 5.5		

^a^ Results are presented in the form of Mean ± SD (*n* = 5).

**Table 3:** Pharmacokinetic parameters of tangeretin (TAN) ^a^.

Pharmacokinetic Parameter	Intravenous	Oral	
Formulation	Solution (*n* = 5)	Suspension (*n* = 6)	Solution (*n* = 5)
Dose (mg·kg^−1^)	10	50	50
*A* (µg·mL^−1^)	2.22 ± 0.48	-	-
*B* (ng·mL^−1^)	254 ± 128	-	-
*α* (10^−2^ × min^−1^)	4.06 ± 1.21	-	-
*β* (10^−3^ × min^−1^)	5.47 ± 1.63	-	-
*V_c_* (L·kg^−1^)	4.20 ± 0.82	-	-
*AUC*_0→*last*_ (10^4^ × min·ng·mL^−1^)	10.5 ± 2.5	1.61 ± 0.34	3.17 ± 1.65
*Cl* (mL·min^−1^·kg^−1^)	94.1 ± 20.2	-	-
*t*_1/2 *λz*_ (min)	166 ± 42	-	-
*MRT*_0→*last*_ (min)	73.3 ± 10.3	260 ± 102	187 ± 73
*MAT* (min)	-	187 ± 102	114 ± 73
*C_max_* (ng·mL^−1^)	2470 ± 557	65.3 ± 20.1	135 ± 46 **
*t_max_* (min)	-	90–120	30–90 **
*Mean F* (%)	-	< 3.05	6.02

^a^ The pharmacokinetic parameters are presented as Mean ± SD; * *p* < 0.05; ** *p* < 0.01 between oral solution and suspension; *A*: hybrid constant *A*; *B*: hybrid constant *B*; *α:* hybrid constant *α*; *β*: hybrid constant *β*; *V_c_*: apparent volume of distribution of the central compartment; *AUC*: area under the plasma concentration-time curve; *Cl*: clearance; *t*_1/2 *λz*_: terminal elimination half-life; *MAT*: mean absorption time; *C_max_*: maximal plasma concentration; *t_max_*: time to maximal concentration; *F*: absolute oral bioavailability.
